# A Short Review on Biomedical Applications of Nanostructured Bismuth Oxide and Related Nanomaterials

**DOI:** 10.3390/ma13225234

**Published:** 2020-11-19

**Authors:** Mattia Bartoli, Pravin Jagdale, Alberto Tagliaferro

**Affiliations:** 1 Department of Applied Science and Technology, Politecnico di Torino, Corso Duca degli Abruzzi 24, 10129 Turin, Italy; mattia.bartoli@polito.it; 2 Italian Institute of Technology, Via Livorno 60, 10144 Torino, Italy; 3 Consorzio Interuniversitario Nazionale per la Scienza e Tecnologia dei Materiali (INSTM), Via G. Giusti 9, 50121 Florence, Italy; pravin.jagdale@iit.it

**Keywords:** bismuth oxide, nanoparticles, radiopacity, chemotherapy, theragnostic

## Abstract

In this review, we reported the main achievements reached by using bismuth oxides and related materials for biological applications. We overviewed the complex chemical behavior of bismuth during the transformation of its compounds to oxide and bismuth oxide phase transitions. Afterward, we summarized the more relevant studies regrouped into three categories based on the use of bismuth species: (i) active drugs, (ii) diagnostic and (iii) theragnostic. We hope to provide a complete overview of the great potential of bismuth oxides in biological environments.

## 1. Introduction

Recently, the concerns for human healthiness have risen day by day. Pharma industries and the research community have committed themselves to improve both the knowledge and on-field real application of newly designed drugs and protocols. Despite the numerous available established treatments, there is still an urgent need to develop new and innovative technologies that could help to delineate tumor margins, identify residual tumor cells and eventually eliminate them [1,2,3].

Furthermore, the development of new antimicrobial agents able to overcome antibiotic resistance rising has become critical [4].

All of these issues have been deeply investigated by nanoscience and material technology. Among all the promising nanostructured or nanosized materials, bismuth-based ones, although rather neglected, are full of unexpressed potential [5].

Bismuth has been used in plenty of applications since the 19th century for the treatment of bacterial infections but its use slowed down in the middle of the 20th century after the reversible bismuth encephalopathy occurred in France and Australia [6,7]. Nonetheless, bismuth-based medical formulations are still being used for stomach issues treatments as bismuth subsalicylate [8,9], colloidal bismuth subcitrate [10,11,12] or bioactive conjugated as ranitidine bismuth citrate [13].

Organo-bismuth derivatives are not the only species of great interest as nanosized oxides and related materials have raised more and more interest in the scientific community due to their cost-effective fabrication processes [14,15], high stability [16] and versatility in terms of morphology [17,18]. Furthermore, the high atomic number of bismuth brings about a high energy radiation attenuation larger than that of lead at an almost negligible risk of toxicity [19]. The combinations of bismuth properties represent a unique chance to exploit singularly or simultaneously cytotoxicity and diagnostic effects. In this paper, we overview the literature providing a synthetic although comprehensive discussion on the main achievements reached by using bismuth oxide and related materials for biological applications. After presenting the main chemical and physical properties of bismuth, we regroup the studies in three main categories dealing respectively with (i) biological activity, (ii) use for the production of biomaterials and diagnostic agents and (iii) production of theragnostic platforms.

## 2. Bismuth Oxide and Related Materials: Productive Strategies

Nowadays, bismuth is mainly produced as a side product of lead streams and could be isolated through the Betterton–Kroll process [20] or through an electrochemical procedure known as Betts electrolytic process [21]. It is obtained in a highly purified form for those applications where it is used as a replacement for lead [22].

Commonly, bismuth is used in form of halide, oxo-halide, nitrate and oxides derivatives. Bismuth halides (BiX_3_, X = F, Cl, Br, I) are generally prepared by treating bismuth oxide in a watery medium by adding the specific HX acid. Bismuth trihalides are bipyramidal molecular species in the gas phase with angle X-Bi-X in the range 96–100° [23]. In the solid phase, they show a variety of different structures based on the halogen present in the crystals. BiF3 shows a pseudo-ionic structure with tricapped trigonal prismatic motive where bismuth atoms are surrounded by nine fluoride atoms, while the other halides show bicapped trigonal prism crystals. Bismuth oxide halides (BiOX) are formed by partial hydrolysis of bismuth halides. BiOF and BiOI can also be made by heating the corresponding halides in the air. BiOX have complex layer lattice structures [24] and, when heated up to 600 °C, BiOCl or BiOBr are decomposed by forming Bi_24_0_31_X_10_ [25].

**Figure 1:**
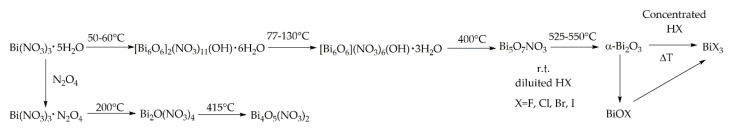
Comprehensive scheme of chemical evolution of Bi(NO_3_)_3_·5 H_2_O.

Moving on, bismuth can easily be produced as bismuth nitrate. Firstly, it is recovered as Bi(NO_3_)_3_·5H_2_O through crystallization after hydrolysis of Bi_2_O_3_ by using concentrated nitric acid. If a diluted acid is used is possible to recover the basic salt BiO(NO_3_). BiO(NO_3_) could be also produced by precipitation treating Bi(NO_3_)_3_·5H_2_O at 150 °C with butanol forming road-like structures as reported by Liu et al. [26]. As clearly enlighten by Briand and Burford [27] the hydrolysis of Bi(NO_3_)_3_·5H_2_O could lead to a plethora of different compounds. Furthermore, several attempts were reported in the literature [28,29,30,31,32] pursuing the thermal oxodehydration of Bi(NO_3_)_3_·5H_2_O with the formation of a series of complex species as summarized in Figure 1.

An interesting study was reported by Tanveer et al. [17] about the transition from Bi(NO_3_)_3_·5H_2_O to Bi_5_O_7_NO_3_ showing how it is possible to isolate a species of Bi_5_O_7_NO_3_ tailored on the surface with β-Bi_2_O_3_.

**Figure 2:**
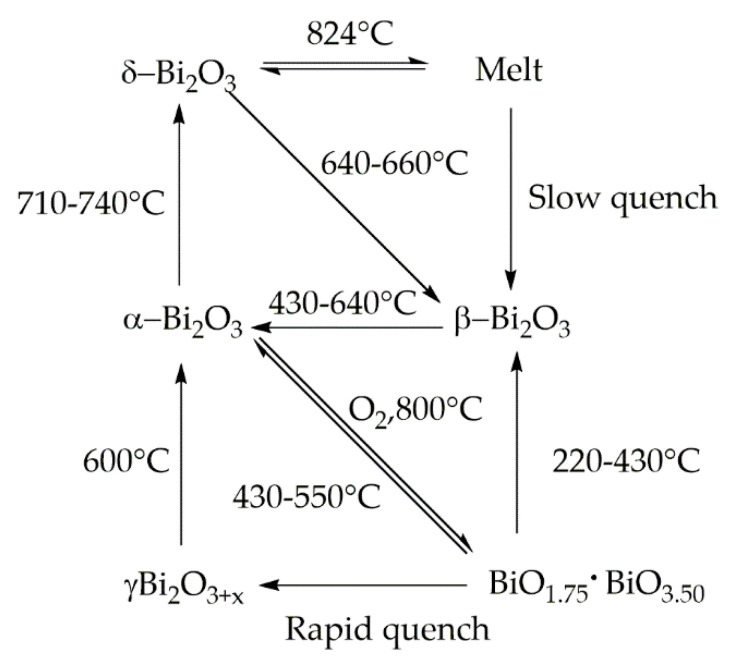
Scheme of phase transition of bismuth oxide.

Bismuth oxides are the other deeply studied class of bismuth compounds and they present four different phases [33] as reported in Figure 2.

At room temperature, monoclinic α-Bi_2_O_3_ is the common stable phase with a polymeric-distorted layered structure composed of pentacoordinate bismuth atoms enclosed into pseudo-octahedral units. At a temperature higher than 710 °C, α phase is converted into the cubic δ phase that has a defective structure with random oxygen vacancies [34]. The β phase and several oxygen-rich forms are closely related to the δ phase. In particular, the vacancy structures of highly defected bismuth oxides some sites filled with O^−2^ together with Bi(III) and Bi (V) sites. Bismuth oxide γ-phase shows also a cubic structure but it is highly unstable and hard to synthesize without supporting it onto other oxides or metallic species [35]. The other two polymorphic metastable bismuth oxide phases are known as the ω phase stable at temperatures higher than 800 °C [36] and the ε phase isolated in 2006 by Cornei and co-workers [37].

Bismuth(V) oxides are less stable than Bi(III) but several studies reported their preparation as lithium [38] or sodium [39] salt derivatives.

Bismuth derivatives were also studied for the production of colloidal phases. Kiran et al. [40] synthesized a bismuth-substituted cobalt ferrite with a nominal formula CoFe_2_−0.1Bi_0.1_O_4_ quite active for the reduction of 4-nitrophenol to 4-aminophenol in a watery solution of sodium borhydride. Metal bismuth nanoparticles were produced by Petsom et al. [41] showing that the size of the nanoparticles can be tuned by adding different amounts of ionic and non-ionic surfactants.

Furthermore, several organometallic species of bismuth such as subgallate [42] and subsalicylate [8] have found use in medical applications that will be more thoroughly discussed in the next sections and briefly summarized in Table 1.

## 3. Bismuth Oxide and Related Materials for Biological Applications

### 3.1. Bismuth Based Nanomaterials as Biological Active Drugs

The first and main point to clarify is about the interaction between bismuth-based materials and living organisms. In 1989, Slikkerveer et al. [44] reported a very comprehensive overview of the toxicity of bismuth species. As clearly emerged, the oral intake of bismuth compounds leads to a significant increase in blood concentrations of bismuth [45] and the amount rose rapidly up to 380 µmL/g [46]. Gavey et al. [47] show how the uptake could be magnified by bismuth citrate soluble species or by the simultaneous administration of cysteine [48]. Lechat et al. [49] reported a study about the administration of bismuth subnitrate showing how poorly or watery insoluble bismuth species decrement the organism uptake. As reported by several studies run on rats using BiCl_3_ [50,51], bismuth binds to high molecular weight metallothionein protein close to those that bind copper cations [52]. Bismuth is excreted by both urine and feces but rats retain up to 10 wt.% of the dose administrated even after 90 days [53].

The in vivo tests suggest that bismuth salts or organometallic derivatives could lead to bioaccumulation and encephalopathy [54]. Stephens et al. [55] used homo- and heteroleptic bismuth(III) thiolates to prove that the bismuth complex surrounding drives the antimicrobial activity of the bismuth species.

Luo et al. [56] reported a study about the in vivo cytotoxicity of different tailored bismuth nanoparticles. The authors produced metallic bare bismuth nanoparticles and tailored their surface with amines, poly(ethyleneglycol), neat and amine tailored silica. The study was run by using HeLa and MG-63 cell lines showing cytotoxicity of bismuth species higher for the HeLa. The authors reported that the non-toxic concentration of bare nanoparticles was attested to 0.5 nM while they induce cellular death at a concentration of up to 50 nM. The functionalization decreased the cytotoxicity of the bare nanoparticles that are more toxic than the other (bare > amine-terminated > silica-coated > poly(ethylene glycol) coated). Coating increased the stability of bismuth inside the cells but decreased its ability to induce oxidative stress.

**Figure 3:**
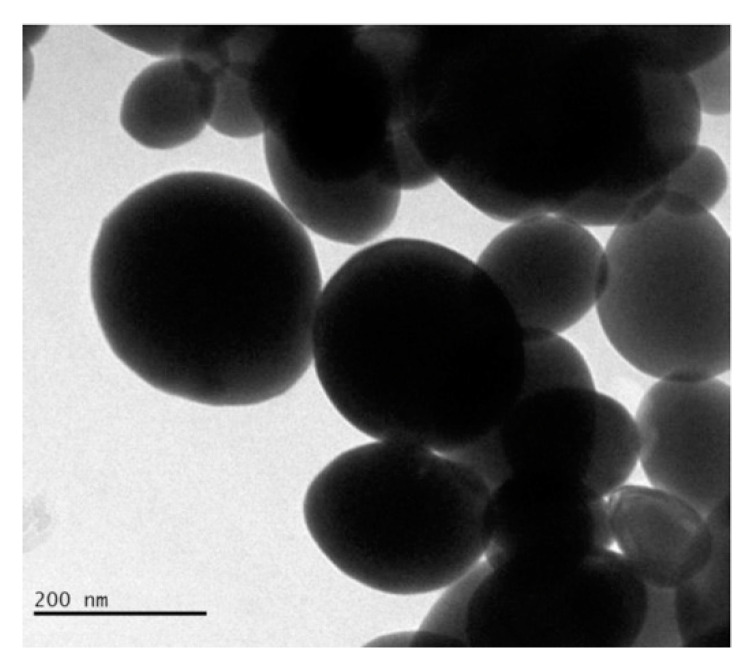
Transmission electronic microscopic capture of bismuth oxide nanoparticles with average diameter ranging from 150 nm to 200 nm. Picture is reprinted with permission from Abudayyak et al. [57].

Abudayyak et al. [57] studied bismuth oxide nanoparticles like the ones shown in Figure 3 regarding their cytotoxicity, genotoxicity, oxidative damage and ability to induce apoptosis in multiple tumoral cell lines (HepG2, NRK, Caco-2, A549).

Authors proved that bismuth oxide nanoparticles differently interacted with different cell lines inducing death through apoptosis in HepG2 and NRK-52E cells and through necrosis in A549 and Caco-2 cells. Among all morphologies, spherical nanoparticles are the most investigated but several studies [58,59,60] have proved that rod-like particles have a higher cellular uptake and transport across intestinal cells. As reported by Truong et al. [61], morphology is a key point to the rational design of biologically active species with cylindrical particles that are the most suitable for tumor accumulation [62]. Among bismuth species, spherical and sponge-like [17] shapes are the most common morphologies but BiONO_3_ [26] could be produced as road-like nanosized particles. Even if this material has been used only as precursors in inorganic synthesis [63] and for the realization of biosensors [26], it could represent an interesting material to improve the bismuth oxide material cellular uptaking.

**Figure 4:**
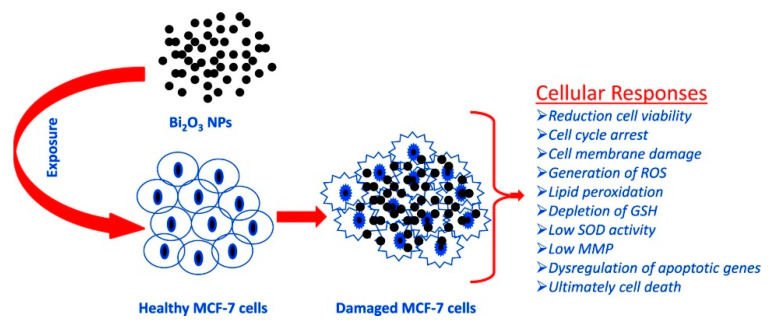
Summary of the process of oxidative stress induced by Bi_2_O_3_ in MCF-7. Picture is reprinted with permission from Ahamed et al. [64].

Ahamed et al. [64] evaluated the effect of Bi_2_O_3_ accordingly to the scheme summarized in Figure 4 by using the MCF-7 cell line.

The authors observed that the bismuth oxide nanoparticles induced apoptotic response in MCF-7cells and suggested this occurs by undermining the regulation of Bcl-2, Bax and caspase-3 genes. Curiously, the authors observed that with the addition of the external antioxidant N-acetyl-cysteine, the bismuth cytotoxicity was almost inhibited. This suggests that the toxicity of bismuth could be tuned by tailoring the composition of the administered formulation.

Considering the study reported by Thomas et al. [65], bismuth-based drugs could show toxicity to human cells even if they are considered sufficiently safe with careful use. The authors individuated the bismuth methylated species as the main cause responsible for the biological damages induced by bismuth administration due to the increased bioavailability.

Genotoxicity of Bi_2_O_3_ was also investigated by Liman [66] showing an unneglectable effect on root cells of *Allium cepa.* Even in combination with Portland cement [67] or other minerals [68], bismuth oxide shows a proved citoxicity and antimicrobial effects during in vivo tests.

**Figure 5:**
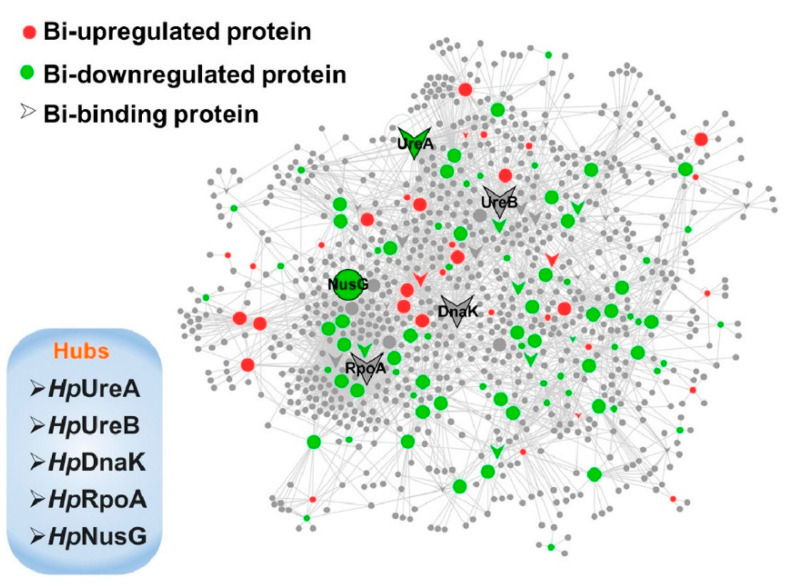
Schematic network depicting bismuth interaction with proteins in *H. pylori.* Proteins are colored and shaped according to their different properties in the network. Adapted with permission from Li et al. [69]. Copyright 2019 American Chemical Society.

Li et al. [69] studied the action mechanism of bismuth-based drugs for treating the Helicobacter Pylori infection by using pharmacology and metalloproteomics approaches. The authors described the efficacy of bismuth-based drugs as a consequence of bismuth ability to interrupt several biological pathways by perturbing the activity of key enzymes as shown in Figure 5. The authors suggested that bismuth materials showed the ability to inhibit metallo-β-lactamase by displacing Zn(II) cofactor and proved useful in the treatment of *Helicobacter pylori* infection.

Liu et al. [70] explained the toxicity of bismuth-related materials as a consequence of the induction of autophagy in kidney cells. In a previous publication, Liu and co-workers [71] evaluated the bismuth oxide nanoparticles cytotoxicity in human embryonic kidney 293 cells. The authors clarified that autophagy bismuth nanoparticles induced cytotoxicity in kidney cells due to the bismuth ion release from nanoparticles. These bismuth ions altered epigenetically the cells through downregulation of DNA methylation of several gene families. The authors also reported that bismuth nanoparticles were uptaken by cells through non-clathrin-regulated endocytosis with an initial internalization into endosomal compartments with further conversion to lysosomes. This step is the key point of the overall bismuth oxides activity because only after the cell internalization the nanoparticles could exploit their features [72]. To facilitate the uptake, several approaches have been proposed but they are counterbalanced by the reduction of bismuth activity due to the stabilization of nanoparticles. For example, Staedler et al. [73] evaluated the cellular uptake and biocompatibility of bismuth ferrite nanoparticles by using A549, NCI-H520 and THP-1 cell lines. The authors showed consolidating results regarding the depletion of cytotoxicity, haemolytic response and biocompatibility enhancement when nanoparticles are coated with a surfactant like poly(ethylenglycol). Tsang et al. [74] tracked the bismuth in *H. pylori* proving that in some cases bismuth materials could enter the cell through the same metallo-protein complexes accountable for the iron uptake.

Moving on from neat bismuth oxides, another interesting bioactive bismuth species is represented by bismuth oxohalides. Gao et al. [75] reported an in vitro study on the cytotoxicity of BiOCl nanosheets in human HaCaT keratinocytes. The authors reported negligible BiOCl cytotoxicity for concentrations lower than 0.5 µg/mL but the appreciable effect on cancerous cells for concentrations ranging from 5 µg/mL of up to 100 µg/mL. The authors related the cytotoxicity of BiOCl with changes in cell morphology and impairment of intracellular organules. Furthermore, BiOCl induced apoptosis through oxidative stress and eventually cells cycle arrest in G0/G1 phase.

**Figure 6:**
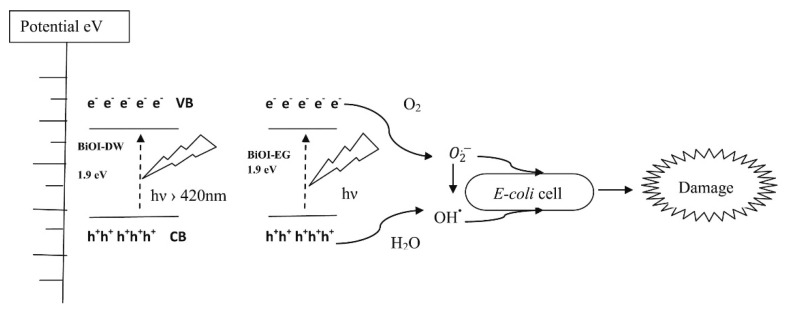
Schematic illustration of energy bands, electron–hole separation and damage mechanism for *E. coli*. Adapted with permission from Jamil et al. [76].

Several proves have been reported on the combination of BiOI photocatalytic activity and antimicrobial effect as described by Jamil et al. [76] and outlined in Figure 6 for the inhibition of *Escherichia coli*.

Authors developed solvothermal template synthesis for the production of flower-like structure as those described for BiOX (X = Cl, Br, I) [15] with a high surface area of up to 410 m^2^/g. Using a catalyst loading of 0.75 g/L in a watery medium, the authors reported a complete photocatalytic inactivation of *E. coli* strain in a concentration of up to 10^5^ CFU/mL.

Similarly, Hsu et al. [77] synthesized gold-doped BiOI nanocomposites through a simple room temperature procedure in an aqueous medium. Gold doped BiOI nanosheets are particularly attractive due to the oxygen vacancies generated in the BiOI lattice structure that increase the oxidation activity. Additionally, the presence of gold nanoparticles enhanced the overall oxidative activity leading to a compound that shows antimicrobial efficacy against *E. coli*, *Klebsiella pneumoniae*, *Salmonella enteritidis*, *Bacillus subtilis* and methicillin-resistant *Staphilococcus aureus*, a methicillin-resistant bacterium. The gold nanoparticles conjugation reduced by up to two-thousand times the minimal inhibitory concentration compared with neat BiOI nanoparticles. The authors also established the pathways of doped BiOI antimicrobial activity that was due to a combined effect of the disruption of the bacterial membrane and the generation of reactive oxygen species. Furthermore, in vivo rabbit model test showed a relevant therapeutic benefit for alleviating corneal *S.aureus* infection without causing inflammatory tissue responses.

Yang et al. [78] reported a study on phototherapy ablation of rabbit orthotopic tumors by using non-stoichiometric BiPO_4−x_ nanoparticles. Through near-infrared light absorption, these oxygen defective structures *promoted* hyperthermia together with the formation of reactive oxygen species. Consequently, they were tested for photothermal/photodynamic therapy in vivo using rabbit as the macroanimal model.

Direct effects of bismuth oxide and related materials on cell viability are not the only appreciable strategies that could base on these materials. Bismuth oxides could be used as effective radiosensitizers species. A radiosensitizer is a chemical that increases the radiation effect on cell viability. These chemotherapy agents are used during radiotherapy in combination with harmful radiation to damage the DNA of cells. As reported by Lawrence [79], radiosensitizing represents the greatest step forward in anticancer treatment and nanoparticle species are one of the most interesting materials for such aim [80]. In 2016, Stewart and co-workers [81] reported the first case of study of bismuth oxide nanoparticles as efficient radiosensitizers on highly radioresistant 9L gliosarcoma cell line. The authors exposed 9 L cells to a bismuth oxide nanoparticle concentration of up to 50 μg/mL achieving a sensitization enhancement of up to 1.5 and 1.3 by using an energy of 125 kV and 10 MV, respectively. Similarly, Liu et al. [82] combined radiotherapy and chemotherapy treatments by administration of mesoporous bismuth litchi-shaped Na_0.2_Bi_0.8_O_0.35_F_1_._91_ as both radiosensitizer and as a nanovehicle for loading and slow-releasing doxorubicin. This bismuth oxide material combined with radiation and doxorubicin showed a remarkable synergistic ability for tumor elimination ability. Farahani et al. [83] combined the bismuth nanoparticles with polymer gel dosimetry technique testing their effect in kilovolt and Megavolt radiation therapy proving the strong energy dependence of dose enhancement.

### 3.2. Bismuth Based Nanomaterials as Additives for the Production of Biomaterials

Bismuth oxides and related materials are quite interesting for all the applications where a high radiopacity together with a good value of biocompatibility is required. Radiopacity is simply defined according to the following equation [84]:

(1) $$I_{\left(x\right)\;}=I_{0}{}^{-\rho \mu_{\left(v\right)}x}$$

where $I_{\left(x\right)}$ is the intensity of the attenuated radiation, $I_{0}$ is the original radiation intensity, *ρ* is the mass density of the material, *µ*_(*ν*)_ is the attenuation coefficient for a fixed radiation frequency and *x* is the length of the travelled path through the material.

For biological applications, radiopacity is measured by using the Hounsfield scale [85] according to the following equation

(2) $$\mathrm{Radiopacity}\;=\;10^{3}\cdot \;\frac{\mu_{\left(v\right)}-\mu_{w}}{\mu_{w}-\mu_{a}}$$

where *µ_w_* is the attenuation coefficient of water and *µ_a_* is the attenuation coefficient of air.

Bismuth based materials have raised great interest in the production of orthodontic cement due to a combination of biocompatibility, radiopacity and antimicrobial effects [86,87].

da Silveira Bueno et al. [88] studied the composition of Bi_2_O_3_ containing Portland cement aiming to obtain an adequate radiopacity for endodontic use. The authors mixed Portland cement with the bismuth oxide with concentrations ranging from 5 wt.% of up to 30 wt.% comparing the results with aluminum foils. Materials reached a radiopacity ranging from 0 to 255 on the Hounsfield scale and a value compatible with shielding applications by using a bismuth concentration of up to 15 wt.%.

Similarly. Chen et al. [89] combined Portland cement not only with Bi_2_O_3_ but also with zirconia. The authors prepared the hybrid bismuth oxide/zirconia compound through a solid-state synthesis at 700 °C for 12 h and mixed it with cement and calcium sulphate. The results showed that the bismuth oxide/zirconia containing cement exploited a greater radiopacity together with the same cell viability of zirconia free one.

Coutinho-Filho et al. [90] evaluated through a histological assessment the subcutaneous connective tissue reactions and the radiopacity of Portland cement mixed with bismuth oxide. The authors reported complete biocompatibility in vivo after 7 and 60 days (no tissue damage observed).

Similarly, several authors reported analogous results for dental repairing applications performed by using Portland and bismuth oxide composites proofing their reliability [91,92].

Furthermore, bismuth could be used for a tissue engineering application as reported by Pazarçeviren et al. [93]. The authors doped a composite made of 45S5 nanobioactive bioglass and graphene oxide with bismuth nanoparticles through a sol–gel methodology. By adding bismuth, authors increased both the composite density and the diametral tensile strength of up to 2.5% retaining cell viability. Additionally, bismuth oxides and related materials could be dispersed into a polymeric matrix to mitigate the effect of harmful radiations during the diagnostic procedures [94,95].

### 3.3. Bismuth Based Nanomaterials as Diagnostic Agents

**Figure 7:**
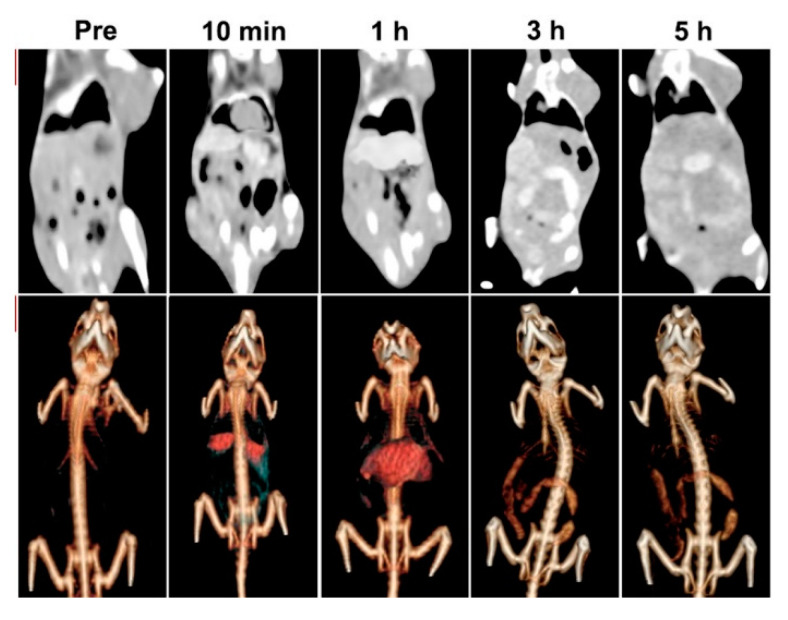
CT images and renderings of CT images of rat after the administration of bismuth modified nanoparticles after different times. Adapted with permission from Bi et al. [96]. Copyright 2018 American Chemical Society.

Bismuth oxides and related materials are also used as contrast agents due to their radiopacity. Bi et al. [96] used poly(ethylenglycol) modified bismuth nanoparticles for applications as multifunctional probes during X-ray computed tomography (CT) and fluorescence imaging. The authors tested the in vivo circulation time and specific accumulation behavior in the liver and intestines by using a CT scan as shown in Figure 7.

Results showed the possible applications of these formulations for target imaging and tracing of the specific areas where bismuth was preferentially accumulated.

Similarly, Swy et al. [97] produced poly(lactic-co-glycolic acid) encapsulated bismuth nanoparticles with an average diameter of less than 40 nm using them as fluorescent probes. The authors achieved a degradation of bismuth-based probes of up to 90% in the acidic and lysosomal-like environment after 24 h while they remained in cytosolic and extracellular-like fluid media.

Several studies have proved the reliability of bismuth oxide as a CT contrast agent with similar or better performances compared with other oxides [98]. Brown et al. [99] developed an ultra-high payload metallic bismuth nanoparticle used as X-ray contrast agents. The authors showed that metallic bismuth nanoparticles will oxidatively decompose to biocompatible Bi(III) based species that are renal excreted after the CT analysis. Dadashi and co-workers [100] combined bismuth nanoparticles together with gold species producing aggregates of up to 40 nm in diameter demonstrating a higher X-ray attenuation in comparison with commercial iodine-based molecules.

**Figure 8:**
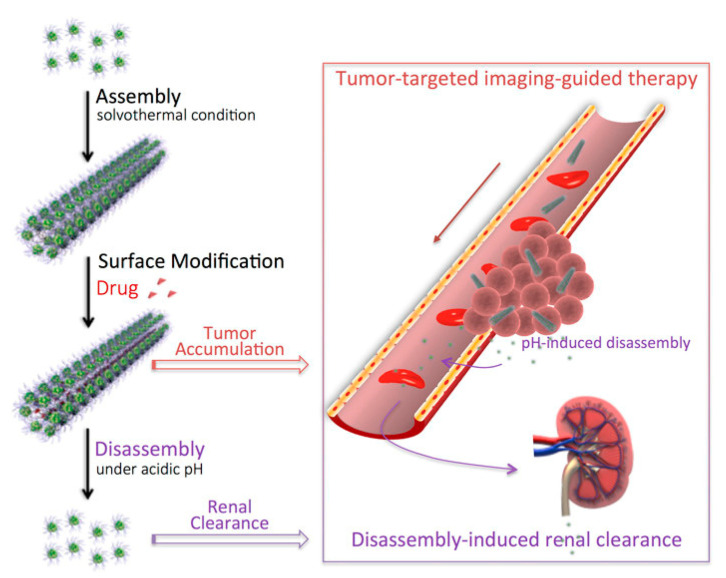
Production and biological pathway of bismuth subcarbonates rods as reported by [101]. Copyright 2018 American Chemical Society.

Hu et al. [101] synthesized a nanostructured (BiO)_2_CO_3_ rod-like material through a solvothermal route and used it as a renal clearable CT contrast agent as shown in Figure 8.

The authors efficiently used the bismuth subcarbonate as a high-resolution CT contrast agent proving that its high aspect ratio actively promoted take-up and retention in the rat tumors tested. The authors also reported the disassembling of the bismuth rods in the acidic microenvironment of tumors enhancing the renal clearance.

Naha et al. [102] reported the production of dextran-coated bismuth/iron oxide nanostructures for magnetic resonance (MR) applications. Results showed a decrement in T2-weighted MR contrast with increasing bismuth content in liver cells. The authors did not observe any cytotoxicity on Hep G2 and BJ5ta cell lines after 24 h incubation with the nanohybrids. Furthermore, the authors ran an in vivo test using mice observing a 2 h circulation time in heart and blood vessels of the bismuth contrast agent. Additionally, this bismuth-based contrast agent was rapidly excreted with urine.

Rivera et al. [103] encapsulated BiOCl into carbon nanostructures and tested it as an agent for X-ray imaging. The authors achieved a high contrast by using a low bismuth loading on nanocarbon (up to 2.7 wt.%) without compromising cell viability. Data enlightened a magnification of up to 500 times of CT resolution compared with traditional iodine-based agents.

BiOCl could be also used as support for the immobilization of aptameric tailored gold nanoparticles as reported by Hsu et al. [104]. This hybrid material showed high peroxidase-like activity and was used for the conversion of Amplex Red proteinic complex to resorufin. According to the authors, this was a very remarkable achievement that proved the robustness of bismuth bioconjugate in proteomic applications.

### 3.4. Bismuth Based Nanomaterials as Active Agents in Theragnostic Platforms

The combination of diagnostic procedures together with a therapeutic protocol is defined as theragnostic and represents the last frontier in advanced treatments [105]. Nanoscale theragnostic is a fast-growing branch of medicinal chemistry for simultaneously monitoring drug release and its distribution, and to evaluate the real-time therapeutic efficacy through a single nanoscale product for both treatment and diagnosis. As reported in the previous sections, bismuth materials are good and efficient contrast agents but could also be exploited for targeted cytotoxicity in vivo. The simultaneous effects herein mentioned led to the development of theragnostic platforms based on bismuth oxides and related materials.

**Figure 9:**
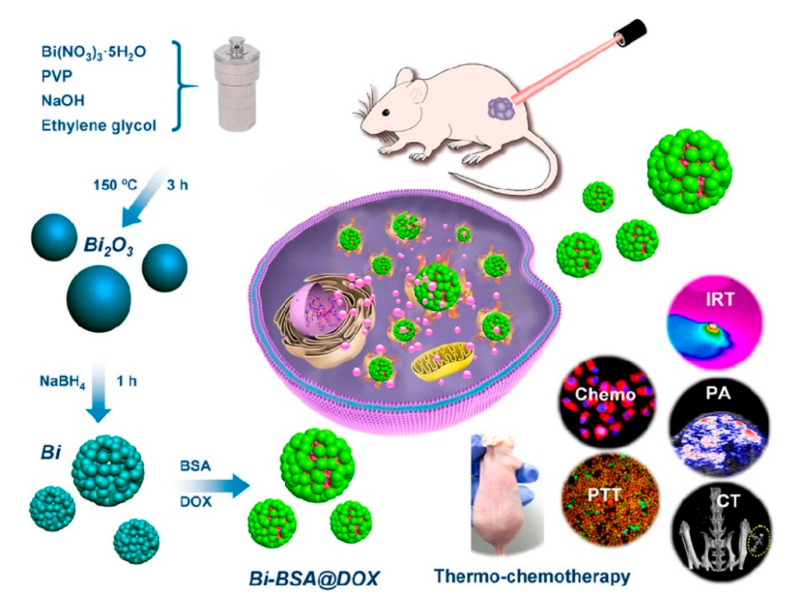
Production and biological action of bismuth oxide nanoraspberries species as reported by Li et al. [106]. Copyright 2018 American Chemical Society.

Li et al. [106] developed a bovine serum albumin modified bismuth oxides nanoraspberries for multimodal imaging and chemo-photothermal combination therapy as summarized in Figure 9.

The authors synthesized the nanoparticles through a watery reduction by using sodium borhydride under pressure at 150 °C for 3 h. The synthesized material showed a surface area of up to 53 m^2^/g and a DOX drug loading of up to 69 wt.% with release occurring upon pH variations. The authors reported the bismuth-based theragnostic agent’s ability to efficiently convert near-infrared light to thermal energy for photothermal ablation of cancer cells. The toxicity studies proved the high biocompatibility without any appreciable toxicity to the mice tested. Additionally, the high radiopacity of bismuth raspberries allows the use of this formulation also during CT analysis. Lu et al. using a similar approach combined the radiopacity of bismuth nanoparticles with photothermal therapy. The authors were able to reach up to 70 °C after 4 min of infrared irradiation showing an enhancement in both CT imaging and in vitro suppression of glioma growth. Xuan et al. [107] prepared bismuth nanoparticles embedded into a nanohydrogel by ultraviolet light-mediated synthesis. The produced materials were combined with DOX and used simultaneously as a contrast agent, as a nanocarrier for drugs and for inducing cell death by thermal ablation. Analogously, Yang et al. [108] produced a bismuth-based CT contrast agent used in photothermal therapy and in ultrasound imaging. They used also several tailored approaches aimed to enhance the theragnostic effects of bismuth preparations. Yu et al. [109] described a thiol capping of bismuth nanoparticles that prevents the unwanted release of bismuth in the organism.

Bismuth oxides and related materials could be also combined with other species. Detappe et al. [110] produced a hybrid material by using ultrasmall silica-based bismuth and gadolinium nanoparticles for dual magnetic resonance and CT imaging while Badrigilan et al. [111] conjugated Bi_2_O_3_ with iron oxides to improve the photothermal behaviour leaving untouched the high bismuth radiopacity.

## 4. Conclusions

Bismuth oxides and related materials show a unique set of features that harvest a relevant interest for plenty of biological applications. Ranging from the production of active drugs to diagnostic agents, bismuth could play a major role in the extension of these productions enhancing the state-of-the-art limit reaching new goals.

While there are plenty of more effective and specific drugs, the combination of radiopacity and tunability of bismuth is quite a unique combination of properties. This represents the starting point for the development of theragnostic platforms that could represent a real game-changing event in the field of advanced medicine. Theragnostic is the field where bismuth could exploit its full potential. Nonetheless, the main unaddressed challenge in the biological application of bismuth is represented by the preparation of an only bismuth-based theragnostic platform where bismuth is simultaneously the contrast agent and the bio-active specie. On this very same topic, a highly speculative but realistic approach could be represented by the realization of a bismuth multilayered particles where the core of bismuth oxide is covered by bismuth oxynitrate tailored with specific biological markers. This hypothesized specie could be the first self-standing mono-element theragnostic preparation where bismuth oxide provides the radiopacity and surface tailoring together with intrinsic defects could provide specific cytotoxicity and drug delivery system.

We strongly believe that the research of new bismuth oxide-based formulations and nanoarchitectures will lead to major breakthroughs with a huge positive impact on humankind’s welfare.

## Figures and Tables

**Table 1:** Summary of main properties of bismuth and related compounds.

Bismuth Species	Advantages	Issues
Metallic bismuth	▪ Easy to synthesized ▪ High size control ▪ Highest concentration of radiopaque atoms	▪ High cytotoxicity for low average size particles ▪ Only spherical shaped ▪ Neat surfaces without any functional groups
Organometallic bismuth	▪ Hydrosoluble ▪ High cellular uptaking ▪ High stability	▪ Low concentration of radiopaque atoms ▪ Fast excretion ▪ Could trespass the hematoencephalic barrier [43]
Bismuth nitrates	▪ High shape tunability ▪ Highly tailoring surface	▪ Fast hydrolysis in watery phase under mild conditions ▪ Difficult to isolated as pure compounds ▪ Difficult to predict the correct active species
Bismuth halide and oxohalides	▪ Easily synthesizable ▪ Photocatalytic activity	▪ Oxidizable ▪ Hygroscopic ▪ Highly acidic
Bismuth oxides	▪ Highly stable ▪ Easy to synthesized ▪ High size control ▪ Poor cytotoxicity ▪ Good cellular uptake ▪ Photocatalytic activity	▪ Highly hydrophobic ▪ Phase impurities ▪ Surface defects

## References

[1] Girish G., Finlay K., Fessell D., Pai D., Dong Q., Jamadar D. (2012). Imaging review of skeletal tumors of the pelvis malignant tumors and tumor mimics. Sci. World J..

[2] Boone C.W., Kelloff G.J., Malone W.E. (1990). Identification of candidate cancer chemopreventive agents and their evaluation in animal models and human clinical trials: A review. Cancer Res..

[3] Tannock I. (1978). Cell kinetics and chemotherapy: A critical review. Cancer Treat. Rep..

[4] Zaman S.B., Hussain M.A., Nye R., Mehta V., Mamun K.T., Hossain N. (2017). A review on antibiotic resistance: Alarm bells are ringing. Cureus.

[5] Shahbazi M.-A., Faghfouri L., Ferreira M.P., Figueiredo P., Maleki H., Sefat F., Hirvonen J., Santos H.A. (2020). The versatile biomedical applications of bismuth-based nanoparticles and composites: Therapeutic, diagnostic, biosensing, and regenerative properties. Chem. Soc. Rev..

[6] Monseu G., Struelens M., Roland M. (1976). Bismuth encephalopathy. Acta Neurol. Belg..

[7] Buge A., Supino-Viterbo V., Rancurel G., Pontes C. (1981). Epileptic phenomena in bismuth toxic encephalopathy. J. Neurol. Neurosurg. Psychiatry.

[8] DuPont H.L., Ericsson C.D., Johnson P.C., Bitsura J.A.M., DuPont M.W., de la Cabada F.J. (1987). Prevention of travelers’ diarrhea by the tablet formulation of bismuth subsalicylate. JAMA.

[9] Bierer D.W. (1990). Bismuth subsalicylate: History, chemistry, and safety. Rev. Infect. Dis..

[10] Wagstaff A.J., Benfield P., Monk J.P. (1988). Colloidal bismuth subcitrate. Drugs.

[11] Goodwin C., Marshall B., Blincow E., Wilson D., Blackbourn S., Phillips M. (1988). Prevention of nitroimidazole resistance in Campylobacter pylori by coadministration of colloidal bismuth subcitrate: Clinical and in vitro studies. J. Clin. Pathol..

[12] Wieriks J., Hespe W., Jaitly K., Koekkoek P., Lavy U. (1982). Pharmacological properties of colloidal bismuth subcitrate (CBS, DE-NOL). Scand. J. Gastroenterol. Suppl..

[13] Canena J., Reis J., Pinto A., Santos A., Leitao J., Pinheiro T., Quina M. (1998). Distribution of bismuth in the rat after oral dosing with ranitidine bismuth citrate and bismuth subcitrate. J. Pharm. Pharm..

[14] Barrera-Mota K., Bizarro M., Castellino M., Tagliaferro A., Hernández A., Rodil S.E. (2015). Spray deposited β-Bi_2_O_3_ nanostructured films with visible photocatalytic activity for solar water treatment. Photochem. Photobiol. Sci..

[15] Jagdale P., Castellino M., Marrec F., Rodil S.E., Tagliaferro A. (2014). Nano sized bismuth oxy chloride by metal organic chemical vapour deposition. Appl. Surf. Sci..

[16] Meitl M.A., Dellinger T.M., Braun P.V. (2003). Bismuth–Ceramic Nanocomposites with Unusual Thermal Stability via High-Energy Ball Milling. Adv. Funct. Mater..

[17] Gadhi T.A., Hernández S., Castellino M., Jagdale P., Husak T., Hernández-Gordillo A., Tagliaferro A., Russo N. (2019). Insights on the role of β-Bi_2_O_3_/Bi_5_O_7_NO_3_ heterostructures synthesized by a scalable solid-state method for the sunlight-driven photocatalytic degradation of dyes. Catal. Today.

[18] Gadhi T.A., Hernández-Gordillo A., Bizarro M., Jagdale P., Tagliaferro A., Rodil S.E. (2016). Efficient α/β-Bi_2_O_3_ composite for the sequential photodegradation of two-dyes mixture. Ceram. Int..

[19] Singh N., Singh K.J., Singh K., Singh H. (2004). Comparative study of lead borate and bismuth lead borate glass systems as gamma-radiation shielding materials. Nucl. Instrum. Methods Phys. Res. Sect. B.

[20] Mallaley K., Morris D. (1989). Analysis of the Betterton-Kroll Process: The Removal of Bismuth from Lead Bullion. Primary and Secondary Lead Processing.

[21] González-Domínguez J., Peters E., Dreisinger D. (1991). The refining of lead by the Betts process. J. Appl. Electrochem..

[22] Zhou Y., Dong F., Jin S. (2018). Bismuth: Advanced Applications and Defects Characterization.

[23] Berry C.R. (1952). Electron diffraction from small crystals. Phys. Rev..

[24] Zhao L., Zhang X., Fan C., Liang Z., Han P. (2012). First-principles study on the structural, electronic and optical properties of BiOX (X = Cl, Br, I) crystals. Phys. B.

[25] Greenwood N.N., Earnshaw A. (2012). Chemistry of the Elements.

[26] Liu G.-Q., Zhong H., Li X.-R., Yang K., Jia F.-F., Cheng Z.-P., Zhang L.-L., Yin J.-Z., Guo L.-P., Qian H.-Y. (2017). Research on nonenzymatic electrochemical sensor using HO-BiONO3 nanocomposites for glucose detection. Sens. Actuators B.

[27] Briand G.G., Burford N. (1999). Bismuth compounds and preparations with biological or medicinal relevance. Chem. Rev..

[28] Lu B., Zhu Y. (2014). Synthesis and photocatalysis performances of bismuth oxynitrate photocatalysts with layered structures. PCCP.

[29] Yang Y., Liang H., Zhu N., Zhao Y., Guo C., Liu L. (2013). New type of [Bi_6_O_6_(OH)_3_](NO_3_)_3_·1.5H_2_O sheets photocatalyst with high photocatalytic activity on degradation of phenol. Chemosphere.

[30] Zahariev A., Kaloyanov N., Girginov C., Parvanova V. (2012). Synthesis and thermal decomposition of [Bi_6_O_6_ (OH)_2_](NH_2_C_6_H_4_SO_3_)_4_. Thermochim. Acta.

[31] Kodama H. (1994). Synthesis of a new compound, Bi_5_O_7_NO_3_, by thermal decomposition. J. Solid State Chem..

[32] Yu S., Zhang G., Gao Y., Huang B. (2011). Single-crystalline Bi_5_O_7_NO_3_ nanofibers: Hydrothermal synthesis, characterization, growth mechanism, and photocatalytic properties. J. Colloid Interface Sci..

[33] Levin E.M., Roth R.S. (1964). Polymorphism of bismuth sesquioxide. I. Pure Bi_2_O_3_. J. Res. Natl. Bur. Stand. Sect. A Phys. Chem..

[34] Lei B., Cui W., Sheng J., Wang H., Chen P., Li J., Sun Y., Dong F. (2020). Synergistic effects of crystal structure and oxygen vacancy on Bi_2_O_3_ polymorphs: Intermediates activation, photocatalytic reaction efficiency, and conversion pathway. Sci. Bull..

[35] Bruton T., Brice J., Hill O., Whiffin P. (1974). The flux growth of some γ-Bi_2_O_3_ crystals by the top seeded technique. J. Cryst. Growth.

[36] Gualtieri A., Immovilli S., Prudenziati M. (1997). Powder X-ray diffraction data for the new polymorphic compound ω-Bi_2_O_3_. Powder Diffr..

[37] Cornei N., Tancret N., Abraham F., Mentré O. (2006). New ε-Bi_2_O_3_ metastable polymorph. Inorg. Chem..

[38] Kumada N., Takahashi N., Kinomura N., Sleight A. (1996). Preparation and crystal structure of a new lithium bismuth oxide: LiBiO_3_. J. Solid State Chem..

[39] Zhang T., Ding Y., Tang H. (2015). Generation of singlet oxygen over Bi (V)/Bi (III) composite and its use for oxidative degradation of organic pollutants. Chem. Eng. J..

[40] Kiran V.S., Sumathi S. (2017). Comparison of catalytic activity of bismuth substituted cobalt ferrite nanoparticles synthesized by combustion and co-precipitation method. J. Magn. Magn. Mater..

[41] Petsom K., Kopwitthaya A., Horphathum M., Kaewkhao J., Sangwaranatee N. (2018). The effect of additive chemicals on synthesis of bismuth nanoparticles. Mater. Today Proc..

[42] Tramontina V.A., Machado M.A.N., Filho G.d.R.N., Kim S.H., Vizzioli M.R., Toledo S. (2002). Effect of bismuth subgallate (local hemostatic agent) on wound healing in rats. Histological and histometric findings. Braz. Dent. J..

[43] Pamphlett R., Stoltenberg M., Rungby J., Danscher G. (2000). Uptake of bismuth in motor neurons of mice after single oral doses of bismuth compounds. Neurotoxicol. Teratol..

[44] Slikkerveer A., de Wolff F.A. (1989). Pharmacokinetics and toxicity of bismuth compounds. Med. Toxicol. Advers. Drug Exp..

[45] Nwokolo C.U., Gavey C.J., Smith J.T.L., Pounder R.E. (1989). The absorption of bismuth from oral doses of tripotassium dicitrato bismuthate. Aliment. Pharm. Ther..

[46] Conso F. (1975). Bismuth Sanguin Et Urinaire Apres Traitement Bref Par Differents Sels Insolubles De Bismuth. Eur. J. Toxicol..

[47] Gavey C., Szeto M.L., Nwokolo C., Sercombe J., Pounder R. (1989). Bismuth accumulates in the body during treatment with tripotassium dicitrato bismuthate. Aliment. Pharm. Ther..

[48] Chaleil D. (1979). Augmentation des concentrations sanguines de bismuth par la cystéine chez le rat. Thérapie.

[49] Lechat P., Majoie B., Levillain R., Cluzan R., Deleau D. (1964). Étude de la toxicité à court terme de l’association sous-nitrate de bismuth et sorbitol. Therapie.

[50] Szymanska J.A., Mogilnicka E.M., Kaszper B.W. (1977). Binding of bismuth in the kidney of the rat. The role of metallothionein-like proteins. Biochem. Pharmacol..

[51] Szymanska J.A., Piotrowski J.K. (1980). Studies to identify the low molecular weight bismuth-binding proteins in rat kidney. Biochem. Pharm..

[52] Żelazowski A.J., Piotrowski J.K. (1980). Mercury-binding, copper-zinc proteins from rat kidney. Amino acid composition, molecular weight and metal content. Biochim. Et Biophys. Acta (Bba)-Protein Struct..

[53] Chaleil D., Regnault J., Allain P., Motta R., Raynaud G. (1988). Action d’une flore microbienne méthanogène d’origine humaine sur l’absorption et la fixation du bismuth chez le rat. Ann. Pharm. Fr..

[54] Buge A. (1974). 20 observations d’encéphalopathies aiguës avec myoclonies au cours de traitements oraux par les sels de bismuth. Ann. Méd. Interne.

[55] Stephens L.J., Munuganti S., Duffin R.N., Werrett M.V., Andrews P.C. (2020). Is Bismuth Really the “Green” Metal? Exploring the Antimicrobial Activity and Cytotoxicity of Organobismuth Thiolate Complexes. Inorg. Chem..

[56] Luo Y., Wang C., Qiao Y., Hossain M., Ma L., Su M. (2012). In vitro cytotoxicity of surface modified bismuth nanoparticles. J. Mater. Sci. Mater. Med..

[57] Abudayyak M., Öztaş E., Arici M., Özhan G. (2017). Investigation of the toxicity of bismuth oxide nanoparticles in various cell lines. Chemosphere.

[58] Banerjee A., Qi J., Gogoi R., Wong J., Mitragotri S. (2016). Role of nanoparticle size, shape and surface chemistry in oral drug delivery. J. Control. Release.

[59] Mitragotri S. (2009). In drug delivery, shape does matter. Pharm. Res..

[60] Christian D.A., Cai S., Garbuzenko O.B., Harada T., Zajac A.L., Minko T., Discher D.E. (2009). Flexible filaments for in vivo imaging and delivery: Persistent circulation of filomicelles opens the dosage window for sustained tumor shrinkage. Mol. Pharm..

[61] Truong N.P., Whittaker M.R., Mak C.W., Davis T.P. (2015). The importance of nanoparticle shape in cancer drug delivery. Expert Opin. Drug Deliv..

[62] Dickerson E.B., Dreaden E.C., Huang X., El-Sayed I.H., Chu H., Pushpanketh S., McDonald J.F., El-Sayed M.A. (2008). Gold nanorod assisted near-infrared plasmonic photothermal therapy (PPTT) of squamous cell carcinoma in mice. Cancer Lett..

[63] Zhou C., Cao J., Lin H., Xu B., Huang B., Chen S. (2015). Controllable synthesis and photocatalytic activity of Ag/BiOI based on the morphology effect of BiOI substrate. Surf. Coat. Technol..

[64] Ahamed M., Akhtar M.J., Khan M.A.M., Alrokayan S.A., Alhadlaq H.A. (2019). Oxidative stress mediated cytotoxicity and apoptosis response of bismuth oxide (Bi_2_O_3_) nanoparticles in human breast cancer (MCF-7) cells. Chemosphere.

[65] Thomas F., Bialek B., Hensel R. (2012). Medical use of bismuth: The two sides of the coin. J. Clin. Toxicol..

[66] Liman R. (2013). Genotoxic effects of Bismuth (III) oxide nanoparticles by Allium and Comet assay. Chemosphere.

[67] Zeferino E., Bueno C.S., Oyama L., Ribeiro D. (2010). Ex vivo assessment of genotoxicity and cytotoxicity in murine fibroblasts exposed to white MTA or white Portland cement with 15% bismuth oxide. Int. Endod. J..

[68] Camilleri J., Montesin F.E., Papaioannou S., McDonald F., Pitt Ford T.R. (2004). Biocompatibility of two commercial forms of mineral trioxide aggregate. Int. Endod. J..

[69] Li H., Wang R., Sun H. (2019). Systems Approaches for Unveiling the Mechanism of Action of Bismuth Drugs: New Medicinal Applications beyond Helicobacter Pylori Infection. Acc. Chem. Res..

[70] Liu Y., Shen C., Zhang X., Yu H., Wang F., Wang Y., Zhang L.W. (2018). Exposure and nephrotoxicity concern of bismuth with the occurrence of autophagy. Toxicol. Ind. Health.

[71] Liu Y., Zhuang J., Zhang X., Yue C., Zhu N., Yang L., Wang Y., Chen T., Wang Y., Zhang L.W. (2017). Autophagy associated cytotoxicity and cellular uptake mechanisms of bismuth nanoparticles in human kidney cells. Toxicol. Lett..

[72] Reus T.L., Machado T.N., Bezerra A.G., Marcon B.H., Paschoal A.C.C., Kuligovski C., de Aguiar A.M., Dallagiovanna B. (2018). Dose-dependent cytotoxicity of bismuth nanoparticles produced by LASiS in a reference mammalian cell line BALB/c 3T3. Toxicol. Vitr..

[73] Staedler D., Passemard S., Magouroux T., Rogov A., Maguire C.M., Mohamed B.M., Schwung S., Rytz D., Jüstel T., Hwu S. (2015). Cellular uptake and biocompatibility of bismuth ferrite harmonic advanced nanoparticles. Nanomed. Nanotechnol. Biol. Med..

[74] Tsang C.-N., Ho K.-S., Sun H., Chan W.-T. (2011). Tracking Bismuth Antiulcer Drug Uptake in Single Helicobacter pylori Cells. J. Am. Chem. Soc..

[75] Gao X., Zhang X., Wang Y., Wang Y., Peng S., Fan C. (2015). An in vitro study on the cytotoxicity of bismuth oxychloride nanosheets in human HaCaT keratinocytes. Food Chem. Toxicol..

[76] Jamil T.S., Mansor E.S., Azab El-Liethy M. (2015). Photocatalytic inactivation of E. coli using nano-size bismuth oxyiodide photocatalysts under visible light. J. Environ. Chem. Eng..

[77] Hsu C.-L., Li Y.-J., Jian H.-J., Harroun S.G., Wei S.-C., Ravindranath R., Lai J.-Y., Huang C.-C., Chang H.-T. (2018). Green synthesis of catalytic gold/bismuth oxyiodide nanocomposites with oxygen vacancies for treatment of bacterial infections. Nanoscale.

[78] Yang C., Huang W., Gao Y., Liu Z., An N., Mu W., Pan Q., Yang B., Guo C., Han X. (2020). Phototherapy ablation of rabbit orthotopic tumors by non-stoichiometric BiPO4−x nanoparticles. Chem. Eng. J..

[79] Lawrence T.S., Blackstock A.W., McGinn C. (2003). The mechanism of action of radiosensitization of conventional chemotherapeutic agents. Semin. Radiat. Oncol..

[80] Brun E., Sicard-Roselli C. (2016). Actual questions raised by nanoparticle radiosensitization. Radiat. Phys. Chem..

[81] Stewart C., Konstantinov K., McKinnon S., Guatelli S., Lerch M., Rosenfeld A., Tehei M., Corde S. (2016). First proof of bismuth oxide nanoparticles as efficient radiosensitisers on highly radioresistant cancer cells. Phys. Med..

[82] Liu J., Deng Y., Qin X., Li B., Zhang J., Xu Y., Ouyang R., Li Y., Miao Y., Sun Y. (2019). Ultrafast Synthesizing Bismuth Mesoporous Nanolitchi Radiosensitizer Loading High Dose DOX for CT-Guided Enhanced Chemoradiotherapy. Acs Appl. Mater. Interfaces.

[83] Farahani S., Riyahi Alam N., Haghgoo S., Shirazi A., Geraily G., Gorji E., Kavousi N. (2020). The effect of bismuth nanoparticles in kilovoltage and megavoltage radiation therapy using magnetic resonance imaging polymer gel dosimetry. Radiat. Phys. Chem..

[84] McNaught A.D., Wilkinson A. (1997). Attenuation Coefficient.

[85] DenOtter T.D., Schubert J. (2019). Hounsfield Unit. StatPearls [Internet].

[86] Deb S., Abdulghani S., Behiri J. (2002). Radiopacity in bone cements using an organo-bismuth compound. Biomaterials.

[87] Chen F., Liu C., Mao Y. (2010). Bismuth-doped injectable calcium phosphate cement with improved radiopacity and potent antimicrobial activity for root canal filling. Acta Biomater..

[88] Da Silveira Bueno C.E., Zeferino E.G., Manhães L.R.C., Rocha D.G.P., Cunha R.S., De Martin A.S. (2009). Study of the bismuth oxide concentration required to provide Portland cement with adequate radiopacity for endodontic use. Oral Surg. Oral Med. Oral Pathol. Oral Radiol. Endod..

[89] Chen X., Song J., Chen X., Yang H. (2019). X-ray-activated nanosystems for theranostic applications. Chem. Soc. Rev..

[90] Coutinho-Filho T., De-Deus G., Klein L., Manera G., Peixoto C., Gurgel-Filho E.D. (2008). Radiopacity and histological assessment of Portland cement plus bismuth oxide. Oral Surg. Oral Med. Oral Pathol. Oral Radiol. Endod..

[91] Hwang Y.-C., Lee S.-H., Hwang I.-N., Kang I.-C., Kim M.-S., Kim S.-H., Son H.-H., Oh W.-M. (2009). Chemical composition, radiopacity, and biocompatibility of Portland cement with bismuth oxide. Oral Surg. Oral Med. Oral Pathol. Oral Radiol. Endod..

[92] Kim E.-C., Lee B.-C., Chang H.-S., Lee W., Hong C.-U., Min K.-S. (2008). Evaluation of the radiopacity and cytotoxicity of Portland cements containing bismuth oxide. Oral Surg. Oral Med. Oral Pathol. Oral Radiol. Endod..

[93] Pazarçeviren A.E., Tahmasebifar A., Tezcaner A., Keskin D., Evis Z. (2018). Investigation of bismuth doped bioglass/graphene oxide nanocomposites for bone tissue engineering. Ceram. Int..

[94] Jagdale P., Rovere M., Ronca R., Vigneri C., Bernardini F., Calzetta G., Tagliaferro A. (2020). Determination of the X-ray attenuation coefficient of bismuth oxychloride nanoplates in polydimethylsiloxane. J. Mater. Sci..

[95] Mehnati P., Arash M., Akhlaghi P. (2018). Bismuth-silicon and bismuth-polyurethane composite shields for breast protection in chest computed tomography examinations. J. Med. Phys..

[96] Bi H., He F., Dong Y., Yang D., Dai Y., Xu L., Lv R., Gai S., Yang P., Lin J. (2018). Bismuth Nanoparticles with “Light” Property Served as a Multifunctional Probe for X-ray Computed Tomography and Fluorescence Imaging. Chem. Mater..

[97] Swy E.R., Schwartz-Duval A.S., Shuboni D.D., Latourette M.T., Mallet C.L., Parys M., Cormode D.P., Shapiro E.M. (2014). Dual-modality, fluorescent, PLGA encapsulated bismuth nanoparticles for molecular and cellular fluorescence imaging and computed tomography. Nanoscale.

[98] Ghazanfari A., Marasini S., Miao X., Park J.A., Jung K.-H., Ahmad M.Y., Yue H., Ho S.L., Liu S., Jang Y.J. (2019). Synthesis, characterization, and X-ray attenuation properties of polyacrylic acid-coated ultrasmall heavy metal oxide (Bi_2_O_3_, Yb_2_O_3_, NaTaO_3_, Dy_2_O_3_, and Gd_2_O_3_) nanoparticles as potential CT contrast agents. Colloids Surf. A.

[99] Brown A.L., Naha P.C., Benavides-Montes V., Litt H.I., Goforth A.M., Cormode D.P. (2014). Synthesis, X-ray Opacity, and Biological Compatibility of Ultra-High Payload Elemental Bismuth Nanoparticle X-ray Contrast Agents. Chem. Mater..

[100] Dadashi S., Poursalehi R., Delavari H. (2018). Optical and structural properties of oxidation resistant colloidal bismuth/gold nanocomposite: An efficient nanoparticles based contrast agent for X-ray computed tomography. J. Mol. Liq..

[101] Hu X., Sun J., Li F., Li R., Wu J., He J., Wang N., Liu J., Wang S., Zhou F. (2018). Renal-Clearable Hollow Bismuth Subcarbonate Nanotubes for Tumor Targeted Computed Tomography Imaging and Chemoradiotherapy. Nano Lett..

[102] Naha P.C., Al Zaki A., Hecht E., Chorny M., Chhour P., Blankemeyer E., Yates D.M., Witschey W.R.T., Litt H.I., Tsourkas A. (2014). Dextran coated bismuth–iron oxide nanohybrid contrast agents for computed tomography and magnetic resonance imaging. J. Mater. Chem. B.

[103] Rivera E.J., Tran L.A., Hernández-Rivera M., Yoon D., Mikos A.G., Rusakova I.A., Cheong B.Y., Cabreira-Hansen M.d.G., Willerson J.T., Perin E.C. (2013). Bismuth@US-tubes as a potential contrast agent for X-ray imaging applications. J. Mater. Chem. B.

[104] Hsu C.-L., Lien C.-W., Wang C.-W., Harroun S.G., Huang C.-C., Chang H.-T. (2016). Immobilization of aptamer-modified gold nanoparticles on BiOCl nanosheets: Tunable peroxidase-like activity by protein recognition. Biosens. Bioelectron..

[105] Pene F., Courtine E., Cariou A., Mira J.-P. (2009). Toward theragnostics. Crit. Care Med..

[106] Li Z., Hu Y., Miao Z., Xu H., Li C., Zhao Y., Li Z., Chang M., Ma Z., Sun Y. (2018). Dual-stimuli responsive bismuth nanoraspberries for multimodal imaging and combined cancer therapy. Nano Lett..

[107] Xuan Y., Song X.-L., Yang X.-Q., Zhang R.-Y., Song Z.-Y., Zhao D.-H., Hou X.-L., An J., Zhang X.-S., Zhao Y.-D. (2019). Bismuth particles imbedded degradable nanohydrogel prepared by one-step method for tumor dual-mode imaging and chemo-photothermal combined therapy. Chem. Eng. J..

[108] Yang C., Guo C., Guo W., Zhao X., Liu S., Han X. (2018). Multifunctional Bismuth Nanoparticles as Theranostic Agent for PA/CT Imaging and NIR Laser-Driven Photothermal Therapy. ACS Appl. Nano Mater..

[109] Yu N., Wang Z., Zhang J., Liu Z., Zhu B., Yu J., Zhu M., Peng C., Chen Z. (2018). Thiol-capped Bi nanoparticles as stable and all-in-one type theranostic nanoagents for tumor imaging and thermoradiotherapy. Biomaterials.

[110] Detappe A., Thomas E., Tibbitt M.W., Kunjachan S., Zavidij O., Parnandi N., Reznichenko E., Lux F., Tillement O., Berbeco R. (2017). Ultrasmall Silica-Based Bismuth Gadolinium Nanoparticles for Dual Magnetic Resonance–Computed Tomography Image Guided Radiation Therapy. Nano Lett..

[111] Badrigilan S., Shaabani B., Gharehaghaji N., Mesbahi A. (2019). Iron oxide/bismuth oxide nanocomposites coated by graphene quantum dots: “Three-in-one” theranostic agents for simultaneous CT/MR imaging-guided in vitro photothermal therapy. Photodiagn. Photodyn. Ther..

